# Decreased expression of KGF/FGF7 and its receptor in pathological hypopigmentation

**DOI:** 10.1111/jcmm.12411

**Published:** 2014-10-14

**Authors:** Valeria Purpura, Flavia Persechino, Francesca Belleudi, Cristina Scrofani, Salvatore Raffa, Severino Persechino, Maria Rosaria Torrisi

**Affiliations:** a Istituto Pasteur-Fondazione Cenci Bolognetti, Dipartimento di Medicina Clinica e Molecolare, Sapienza Universita‘ di RomaRoma, Italy; b Azienda Ospedaliera S. AndreaRome, Italy; c NESMOS, Unità di Dermatologia, Sapienza Universita‘ di RomaRome, Italy

To the Editor:

The molecular mechanisms and cellular pathways involved in cutaneous pigmentation, as well as the crucial role played by the epidermal keratinocytes in the process, are just starting to be elucidated. In fact, a number of recent studies from different authors including our group have pointed out that the uptake by keratinocytes of the melanosomes released by the melanocytes occurs through phagocytic ingestion and is regulated by the activity of some receptors, such as protease-activated receptor-2 (PAR-2) and keratinocyte growth factor receptor/fibroblast growth factor receptor 2b (KGFR/FGFR2b), followed by actin cytoskeleton reorganization [1–6]. Dermal fibroblasts are known to participate in this complex cellular interplay controlling pigmentation through the modulated secretion of growth factors [7], some of them acting directly on the melanocytes and stimulating the melanogenesis, such as stem cell factor and basic fibroblast growth factor [8], while others promoting the melanosome phagocytic uptake by the keratinocytes, as occurring in the case of keratinocyte growth factor/fibroblast growth factor 7 (KGF/FGF7): in this context, in fact, we have proposed that the paracrine growth factor KGF, released from dermal fibroblasts, promotes melanosome transfer through binding to and activation of its tyrosine kinase receptor KGFR, expressed on the keratinocytes, but not on melanocytes or fibroblasts: the receptor signalling recruits and activates phospholipase Cγ, an essential player of the phagocytic process [5]. In mouse keratinocytes, KGFR stimulates melanosome uptake also through a signalling pathway involving integrin-linked kinase and RAS-related C3 botulinum toxin substrate 1 (Rac1) [9], suggesting the existence of a crosstalk between KGFR and integrins. In addition, the contribution of increased expression of KGF/FGF7 in hyperpigmented solar lentigo lesions has been demonstrated [10].

**Fig. 1:**
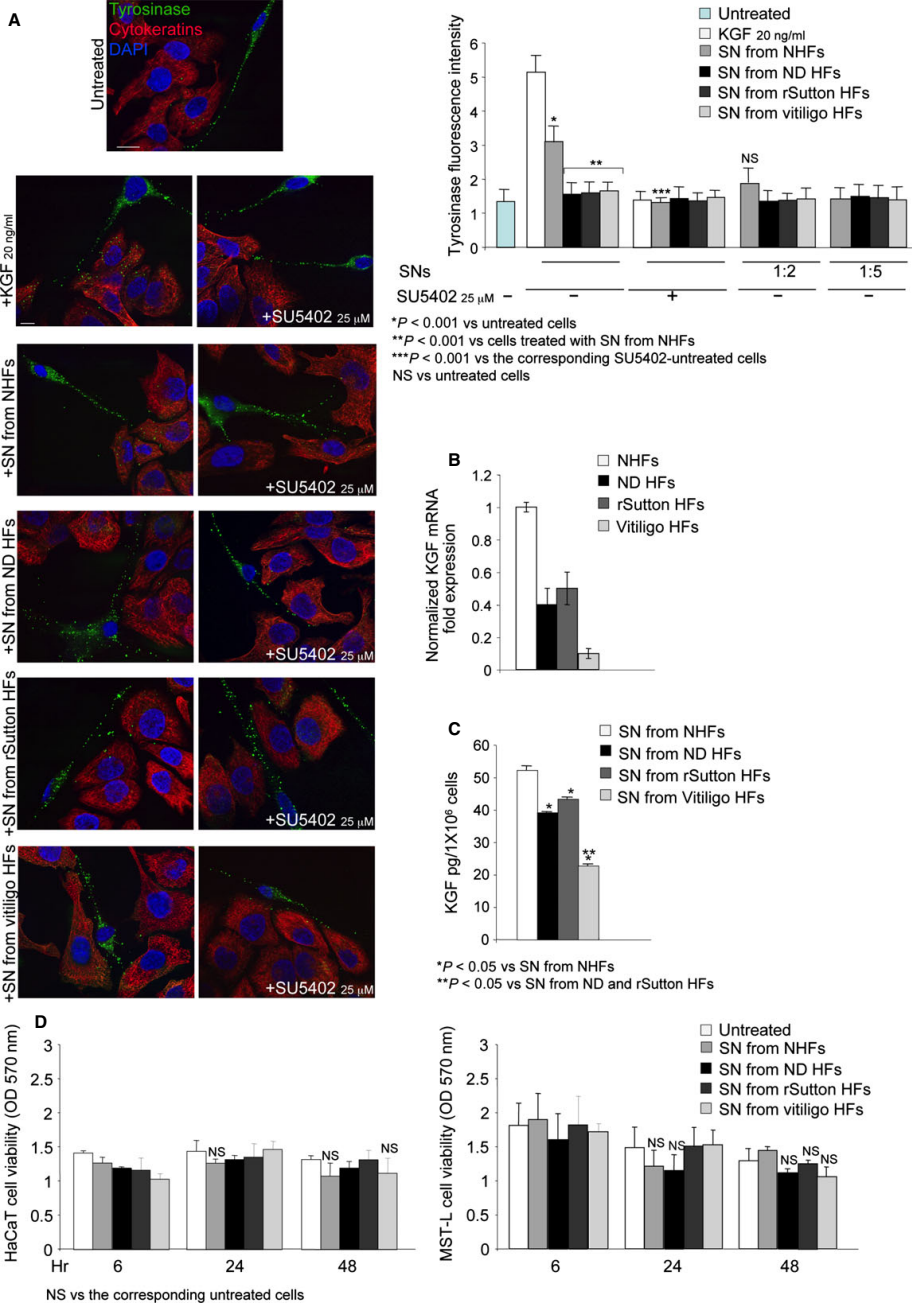
Decreased expression and release of KGF from hypopigmentary lesional fibroblasts leads to reduced melanosome transfer. (**A**) Cocultures of MST-L melanoma cells and HaCaT keratinocytes were stimulated with KGF or with SNs (undiluted or diluted 1:2 or 1:5) from NHFs or lesional HFs. Immunofluorescence shows a significant decrease of the fluorescent tyrosinase-positive dots, corresponding to transferred melanosomes, in the pancytokeratin-positive keratinocytes upon stimulation with SNs from lesional HFs with respect to the treatment with the SN from NHFs or with KGF. The KGFR inhibitor SU5402 blocks the melanosome uptake. Quantitation of tyrosinase fluorescence intensity and Student's *t*-test were performed as reported in Data S1; bars: 10 μm. (**B**) Real-time RT-PCR reveals a decreased KGF mRNA expression in HFs from the lesional samples compared with the control NHFs. (**C**) Quantitation of the released KGF protein by ELISA test performed on the SNs shows that KGF levels in the SNs from lesional samples are significantly decreased with respect to control fibroblasts. Results represent the mean values ± SD. Mann–Whitney test was performed and significance level has been defined as described in Data S1. (**D**) MTT test shows that none of the treatments with SNs is cytotoxic for the cells up to 48 hrs. Results represent the mean values ± SD and Student's *t*-test was performed as reported in Data S1.

Hypopigmentary disorders such as vitiligo and nevus depigmentosus (ND) are characterized by a local or diffuse altered skin pigmentation. In addition, a hypopigmented halo surrounding a central benign melanocytic nevus is the hallmark of the Sutton's nevus. Although the loss of melanocytes is considered the main factor leading to skin colour impairment in such disorders, an altered melanogenesis or a reduced melanosome transfer from melanocytes to keratinocytes is also involved. In fact, it has been proposed that the differential feature of the ND disorder, compared with vitiligo, is the presence of melanocytes with defective melanosome transfer [11,12]. Given the crucial role of the secreted KGF/FGF7 in the modulation of the melanosome uptake by keratinocytes [2,4,9] and taking advantage of our *in vitro* models of melanosome transfer [5], we first investigated here the efficiency of melanosome transfer in the above-mentioned hypopigmentation conditions as well as the ability of supernatants (SNs) collected from primary cultured human dermal fibroblasts, derived from the different lesional skin samples or from healthy donors as described in the Data S1, to stimulate the process. To this aim, the human melanoma cell line MST-L was cocultured with human HaCaT keratinocytes at a seeding ratio of 1:20, as previously described [2,5], serum starved for 12 hrs and incubated for 6 hrs at 37°C with the SNs (undiluted or diluted 1:2 or 1:5) obtained from fibroblasts derived from normal skin (NHFs) or from a nevus depigmentosus lesion (ND HFs), from a vitiligo biopsy (vitiligo HFs) or from the hypopigmented regression area surrounding a Sutton's nevus (rSutton HFs). As positive control, stimulation of the melanosome transfer was induced treating the cocultures with KGF. Double immunofluorescence analysis was performed with anti-tyrosinase polyclonal antibodies, to visualize melanosomes, and anti-pancytokeratin monoclonal antibody, to identify the keratinocytes. Quantitation of tyrosinase fluorescence intensity in the cytosolic area of the keratinocytes, performed as described [5], showed a significant decrease of the tyrosinase-positive dots upon stimulation with lesional-derived SNs with respect to that observed under treatment with SN from NHFs (Fig. 1A, upper panels).

To evaluate if the effects of the various SNs would be ascribed, at least in part, to the presence of KGFR/FGFR2b ligands released in the fibroblast culture medium, as previously demonstrated in previous papers from our group [13,14], addition of the specific FGFR2 tyrosine kinase inhibitor SU5402 was also performed: significant inhibition of the melanosome uptake was found only when the inhibitor was added to the SN from NHFs or to the KGF-treated cultures (Fig. 1A, lower panels), suggesting a possible deficiency of paracrine KGFR ligands in the pathological lesions. Then, to assess if the reduction of melanosome transfer in response to SNs from lesional fibroblasts would be dependent on an altered expression of KGF, the growth factor mRNA transcript levels were analysed by real-time RT-PCR and normalized with respect to β-actin, showing a clear decrease of KGF mRNA expression in all groups of HFs derived from lesional skin compared with the control NHFs (Fig. 1B). In addition, ELISA test demonstrated that KGF protein levels were significantly decreased in SNs from all lesional HFs compared with NHFs (Fig. 1C). Interestingly, consistent with the mRNA expression data, the KGF released by vitiligo HFs was significantly reduced if compared with that secreted by both ND HFs and rSutton HFs (Fig. 1C). None of the SNs was cytotoxic for the cells at different times of treatment (6, 24 or 48 hrs) when assayed by MTT test (Fig. 1D). Thus, the loss of pigmentation in all the three hypopigmentary conditions could be explained, at least in part, by a reduced expression and secretion of KGF from dermal fibroblasts, which impair the melanosome uptake by the keratinocytes.

**Fig. 2:**
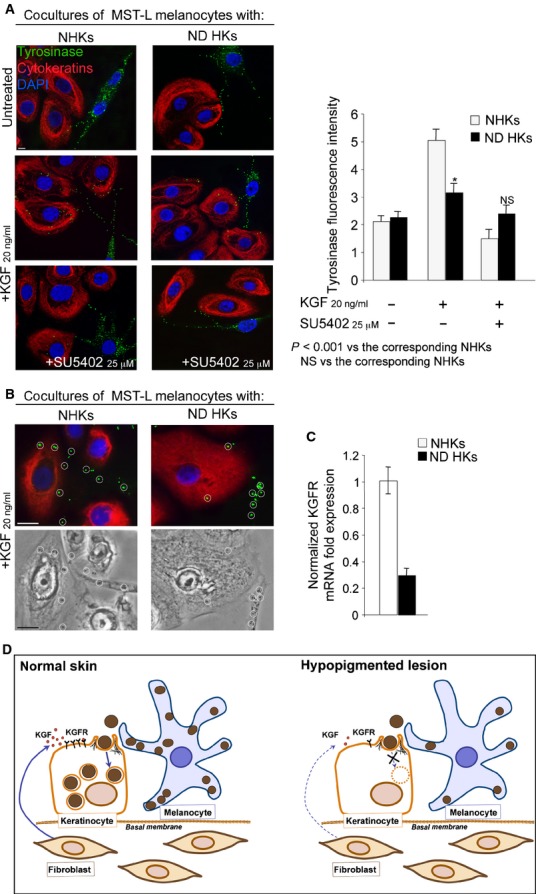
Decreased melanosome uptake ability and KGFR expression in keratinocytes from ND lesion. (**A** and **B**) Cocultures of MST-L melanoma cells with normal human keratinocytes (NHKs) or with keratinocytes derived from the ND lesion (ND HKs) were treated with KGF. Immunofluorescence (**A** and **B**) and phase-contrast (**B**) images show that the tyrosinase-positive dots in ND HKs upon KGF treatment are strongly reduced with respect to those in NHKs (**A** and **B**, circles) and that the addition of SU5402 abolishes the KGF effect; bars: 10 μm. (**C**) Real-time RT-PCR reveals a decreased KGFR mRNA expression in ND HKs compared with NHK control cells. (**D**) Schematic drawing showing the effects of decreased levels of KGF and KGFR on melanosome transfer in hypopigmented lesions.

To evaluate the contribution of the lesional keratinocytes on the inefficient melanosome transfer, we focused our attention on the above ND biopsy, because of the postulated defect of the organelle uptake in such disorder [11,12]. To dissect *in vitro* the process, we cocultured the MST-L melanocytes with primary keratinocytes derived from the ND (ND HKs) or from normal skin, at a seeding ratio of 1:40. Serum starvation and treatment with KGF in the presence or absence of SU5402 were performed as above. The quantitative double immunofluorescence revealed that the KGF-induced increase of the tyrosinase-positive dots in the cytoplasm of ND HKs was much lower compared with NHKs (Fig. 2A, middle panels). Brightfield and phase-contrast microscopy were used to unequivocally demonstrate the decreased melanosome transfer to the lesional keratinocytes (Fig. 2B). Again, the addition of SU5402 was able to abolish the KGF effect in both cocultures (Fig. 2A, lower panels), providing a further evidence of the involvement of KGFR activation and signalling in the process and suggesting a decreased receptor expression in the pathological condition. Therefore, with the aim to analyse the receptor expression, we quantified KGFR transcript levels by real-time RT-PCR and we found a decreased receptor mRNA expression in ND HKs compared with NHK control cells (Fig. 2C). Thus, at least in the ND disorder, low levels of KGFR might significantly contribute to the reduction of KGF-mediated melanosome transfer.

Taken together, our results further support the key roles played, on the melanosome transfer in normal skin, by KGF/FGF7 released by dermal fibroblasts and by its receptor KGFR/FGFR2b expressed and activated on the epidermal keratinocytes (Fig. 2D, cartoon on the left) and suggest a deficient expression of both players (Fig. 2D, cartoon on the right) as an additional pathogenic mechanism involved in hypopigmentary disorders.
