# Supplementary material for: Decreased expression of KGF/FGF7 and its receptor in pathological hypopigmentation
Source: J Cell Mol Med. 2014 Oct 14;18(12):2553–7. doi: 10.1111/jcmm.12411 (PMC4302659; doi:10.1111/jcmm.12411)
Supplement: Supplementary file 1 — Data S1 Supplementary Materials and Methods [file jcmm0018-2553-sd1.doc]

**Supplementary Materials and Methods**

*Patients and tissue samples:* Samples from healthy skin and from a Vitiligo lesion were obtained from patients who underwent surgery or biopsy for various reasons. Specimens from a Nevus Depigmentosus (ND) and from a regression hypopigmented halo area surrounding a Sutton nevus developed within the above ND were collected from the same patient during surgical procedures performed for the excision of the Sutton’s nevus. The samples were obtained from patients attending the Dermatology Unit of the Sant’Andrea Hospital of Rome; all patients were extensively informed and their consent for the investigation was given and collected in written form in accordance with guidelines approved by the management of the Sant'Andrea Hospital.

*Cell cultures and treatments:* The human keratinocyte cell line HaCaT (Boukamp et al., 1988) was cultured in Dulbecco’s modified Eagle medium (DMEM), supplemented with 10% fetal bovine serum (FBS) plus antibiotics. The human melanoma cell line MST-L (kindly provided by Dr. Armando Bartolazzi, Sant'Andrea Hospital, Roma) was cultured in RPMI supplemented with 10% FBS and antibiotics (Cardinali et al. 2005, Belleudi et al. 2011). Primary cultures of human fibroblasts derived from vitiligo (vitiligo HFs), from nevus depigmentosus (ND HFs), from the regression area of the Sutton’s nevus (rSutton HFs) or from healthy skin (NHFs) were also obtained and cultured as described (Raffa et al. 2012). Primary cultures of human keratinocytes derived from the Nevus Depigmentosus (ND HKs) lesion and from healthy skin (NHKs) were obtained as previously described (Cardinali et al. 2005) and maintained in Medium 154-CF (Cascade Biologics, Portland, OR, USA) supplemented with Human Keratinocyte Growth Supplement (HKGS, Cascade Biologics) plus antibiotics and Ca^2+^ 0,03 μM (Cascade Biologics).

In cocultures, primary human keratinocytes and MST-L melanoma cells were seeded at a ratio of 40:1 and were maintained in Medium 154-CF supplemented with HKGS and Ca^2+^ 0.03 μM. Alternatively cocultures of HaCaT and MST-L cells were seeded at a ratio of 20:1 and were maintained in DMEM with 10% FBS and antibiotics.

The supernatants (SNs), obtained from the primary cultured HFs kept in serum-free medium for 48 h, were collected and frozen at -80°C until use.

To analyze the melanosome transfer, all cocultures were serum starved for 12 h at 37°C and incubated with 20 ng/ml KGF (Upstate, Lake Placid, NY, USA) or with SNs for 6 h at 37°C. Both the growth factor concentration and the single time point of treatment have been selected as optimal experimental conditions in previous papers from our group (Cardinali et al. 2005, Belleudi et al. 2011). The SNs were used undiluted or diluted 1:2 or 1:5. For inhibition of KGFR kinase activity, cells were pre-incubated with a specific FGFR2 tyrosine kinase inhibitor, SU5402 (25 μM; Calbiochem, Nottingham, UK) for 1 h before treatment with the growth factor or with the SNs, as previously shown (Belleudi et al. 2011).

*Cell toxicity assay:* To evaluate the cell toxicity in response to treatments with SNs, the MTT assay was used in HaCaT and MST-L cells. Cells were seeded and grown to confluence into 96 -well plates and then treated with undiluted SNs for different times (6, 24 or 48 h). For each time point, cells grown in normal medium without FBS were used as control. Samples were then washed, incubated with 3-(4,5-dimethyltiazol-2-yl)-2,5-dphenyltetrazolium bromide (MTT; Sigma Chemical, St Louis, MO) 5 mg/ml for 2 h at 37°C and then dissolved in dimethyl sulfoxide (Sigma Chemicals). For each sample, the living metabolically active cells were indirectly evaluated measuring the absorbance at 570 nm using the Multiskan® Spectrum spectrofotometer (Thermo Scientific, Waltham, MA, USA). Data obtained from three independent experiment were expressed as mean values ± standard deviation (SD). p values were calculated using Student’s *t* test and significance level has been defined as p<0.05

*Immunofluorescence*: Cocultures, grown on coverslips and incubated as above, were fixed with 4% paraformaldehyde in PBS for 30 min at 25°C, followed by treatment with 0.1 M glycine for 20 min at 25°C and with 0.1% Triton X-100 for an additional 5 min at 25°C to allow permeabilization. Cells were then incubated for 1 h at 25°C with the primary antibodies goat polyclonal anti-tyrosinase (1:50 in PBS; C-19, Santa Cruz Biotechnology, Santa Cruz, CA, USA) and mouse monoclonal anti-pancytokeratin (1:100 in PBS; clone MNF116; DAKO, Carpinteria, CA, USA). The primary antibodies were visualized, after appropriate washing with PBS, using Alexa Fluor 488 conjugated chicken anti-goat IgG (1:1000 in PBS; Molecular Probes, Eugene, OR, USA) and Texas-Red conjugated rabbit anti-mouse IgG (1:200 in PBS; Jackson Immunoresearch Laboratories, West Grove, PA, USA). Nuclei were stained with DAPI (1:10.000 in PBS; Sigma Chemical, St Louis, MO). Coverslips were finally mounted with mowiol for observation. Fluorescence signals were analyzed by conventional fluorescence or by scanning cells in a series of 0.5μm sequential sections with an ApoTome System (Zeiss, Oberkochen, Germany) connected with an Axiovert 200 inverted microscope (Zeiss); image analysis was then performer by the Axiovision software (Zeiss). Quantitative analysis of the tyrosinase fluorescence intensity for cytoplasmic area was performed by the analysis of 100 cells for each sample in 5 different fields randomly taken from three independent experiments and using the KS300 3.0 Image Processing System (Zeiss); results are shown as means ± SD. p values were calculated using Student’s *t* test and significance level has been defined as p<0.05

*Primers:* Oligonucleotide primers for target genes and for the housekeeping gene were chosen with the assistance of the Oligo 5.0 computer program (National Biosciences, Plymouth, MN) and purchased from Invitrogen. The following primers were used: for FGFR2b/KGFR target gene: 5’-CAGGGGTCTCCGAGTATGAA-3 (sense), 5’-TCTAAAGGCAACCTCCGAGA-3’ (anti-sense); for KGF target gene: 5-CACCAGGCAGACAACAGACAT-3 (sense), 5-GTAAGTTCAGTTGCTGTGACGCT-3 (anti-sense); for the β-actin housekeeping gene: 5’-CATCAGCAATGCCTCCTGCAC-3’ (sense), 5’-GTCATGAGTCCTTCCACGATACCAA-3’ (antisense). For each primer pair, we performed no-template control and no-reverse-transcriptase control (RT negative) assays, which produced negligible signals.

*RNA extraction and cDNA synthesis:* RNA was extracted using the TRIzol method (Invitrogen, Carlsbad, CA) according to manufacturer’s instructions and eluted with 0,1% diethylpyrocarbonate (DEPC)-treated water. Total RNA concentration was quantitated by spectrophotometry and the quality was assessed by measuring the optical density ratio at 260/280 nm. RNA samples were stored at -80°C. After denaturation in DEPC-treated water at 70°C for 10 minutes, 1 mg of total RNA was used to reverse transcription using iScript^TM^ cDNA synthesis kit (Bio-Rad Laboratoires, Hercules, CA) according to manufacturer’s instructions.

*PCR amplification and real-time quantitation:* Real-time PCR was performed using the iCycler Real-Time Detection System (iQ5 Bio-Rad) with optimized PCR conditions. The reaction was carried out in 96-well plate using iQ SYBR Green Supermix (Bio-Rad) adding forward and reverse primers for each gene and 1 μl of diluted template cDNA to a final reaction volume of 15 μl. All assays included a negative control and were replicated three times. The thermal cycling program was performed as follows: an initial denaturation step at 95°C for 3 minutes, followed by 45 cycles at 95°C for 10 seconds and 60°C for 30 seconds. Real-time quantitation was performed with the help of the iCycler IQ optical system software version 3.0a (Bio-Rad), according to the manufacturer’s manual. The relative expression of the housekeeping gene was used for standardizing the reaction. The comparative threshold cycle (C_t_) method was applied to calculate the fold changes of expression compared to control cells Results are reported as mean ± standard deviation (SD) from three different experiments in triplicate.

*ELISA:* KGF in the SNs collected from the cultures was quantified using the human KGF ELISA kit (Quantikine®; R&D Systems, Minneapolis, MN) according to the manufacturer’s protocol. A standard curve for each ELISA experiment was prepared from duplicate wells with increasing concentrations of KGF (0-31.2-62.5-125-250-500-1000-2000 pg/ml), using the protocol described above. The results were normalized for the number of cells contained in each sample and were expressed as picograms/1×10^6^ cells. Each sample was analyzed in triplicate. Mann–Whitney test was performed to evaluate significant differences between samples and  *p*<0.05 was considered statistically significant.

**References**

- Belleudi F, Purpura V, Scrofani C *et al* (2011). Expression and signaling of the tyrosine kinase FGFR2b/KGFR regulates phagocytosis and melanosome uptake in human keratinocytes. *FASEB J* 25: 170-81.

- Boukamp P, Petrussevska RT, Breitkreutz D *et al* (1988). Normal keratinization in a spontaneously immortalized aneuploid human keratinocyte cell line. *J Cell Biol* 106: 761-71.

- Cardinali G, Ceccarelli S, Kovacs D *et al* (2005). Keratinocyte growth factor promotes melanosome transfer to keratinocytes. *J Invest Dermatol* 125: 1190-9.

- Raffa S, Leone L, Scrofani C *et al* (2012). Cholesteatoma-associated fibroblasts modulate epithelial growth and differentiation through KGF/FGF7 secretion. *Histochem Cell Biol* 138: 251-69.
